# [Retracted] Effects of FHIT gene on proliferation and apoptosis of osteosarcoma cells

**DOI:** 10.3892/ol.2026.15772

**Published:** 2026-07-20

**Authors:** Zhengfeng Xu, Jiajun Wu, Pan Cai, Xiaoxiao Zhou, Cunguo Yi, Bin Wang

Oncol Lett 17: 877–882, 2019; DOI: 10.3892/ol.2018.9696

Following the publication of the above paper, it was drawn to the Editor's attention by a concerned reader that, regarding the colony formation assay images shown in Fig. 2 on p. 879, the ‘Saos2 group’ and ‘No-load transfection group’ images were strikingly similar, albeit the panels were rotated through 90° relative to each other, such that these data, which were intended to show the results of differently performed experiments, had apparently been derived from the same original source. In addition, upon performing an independent analysis of the data in this paper in the Editorial Office, it came to light that certain of the data in this figure appeared subsequently in a paper in the journal *Archives of Medical Sciences*, and had already appeared in a paper in the journal *PLoS One*, where both articles had been written by different authors at different research institutes.

Owing to the fact that the contentious data in the above article had already been published prior to its submission to *Oncology Letters*, the Editor has decided that this paper should be retracted from the Journal. The authors were asked for an explanation to account for these concerns, but the Editorial Office did not receive a reply. The Editor apologizes to the readership for any inconvenience caused.

